# Endothelin-1 Mediated Decrease in Mitochondrial Gene Expression and Bioenergetics Contribute to Neurodegeneration of Retinal Ganglion Cells

**DOI:** 10.1038/s41598-020-60558-6

**Published:** 2020-02-27

**Authors:** Renuka M. Chaphalkar, Dorota L. Stankowska, Shaoqing He, Bindu Kodati, Nicole Phillips, Jude Prah, Shaohua Yang, Raghu R. Krishnamoorthy

**Affiliations:** 1 North Texas Eye Research Institute, Department of Pharmacology and Neuroscience, UNT Health Science Center, Fort Worth, Texas 76107 United States; 2 Department of Microbiology, Immunology and Genetics, UNT Health Science Center, Fort Worth, Texas 76107 United States; 3 Department of Basic and Translational Sciences, School of Dental Medicine, University of Pennsylvania, Philadelphia, PA 19104 United States

**Keywords:** Visual system, Retina

## Abstract

Endothelin-1 (ET-1) is a vasoactive peptide that is elevated in aqueous humor as well as circulation of primary open angle glaucoma (POAG) patients. ET-1 has been shown to promote degeneration of optic nerve axons and apoptosis of retinal ganglion cells (RGCs), however, the precise mechanisms are still largely unknown. In this study, RNA-seq analysis was used to assess changes in ET-1 mediated gene expression in primary RGCs, which revealed that 23 out of 156 differentially expressed genes (DEGs) had known or predicted mitochondrial function, of which oxidative phosphorylation emerged as the top-most enriched pathway. ET-1 treatment significantly decreased protein expression of key mitochondrial genes including cytochrome C oxidase copper chaperone (COX17) and ATP Synthase, H^+^ transporting, Mitochondrial Fo Complex (ATP5H) in primary RGCs and *in vivo* following intravitreal ET-1 injection in rats. A Seahorse ATP rate assay revealed a significant decrease in the rate of mitochondrial ATP production following ET-1 treatment. IOP elevation in Brown Norway rats showed a trend towards decreased expression of ATP5H. Our results demonstrate that ET-1 produced a decrease in expression of vital components of mitochondrial electron transport chain, which compromise bioenergetics and suggest a mechanism by which ET-1 promotes neurodegeneration of RGCs in glaucoma.

## Introduction

Glaucoma is an optic neuropathy with an approximate prevalence of 60.5 million people worldwide and is projected to reach 111 million by 2040^1,2^.The disease is commonly associated with elevated intraocular pressure (IOP), accompanied by optic nerve degeneration and loss of retinal ganglion cells (RGCs)^3,4^. RGC death via apoptosis is a culminating event in the pathophysiology of glaucoma, stemming from optic nerve axonal injury, leading to visual field loss. Elevated IOP is a major risk factor in primary open-angle-glaucoma and current therapeutic approaches are aimed at lowering IOP with medications, laser treatment, or surgery^5,6^. However in some patients, the progression of the disease continues to occur slowly^7^ despite lowering IOP, hence there is a compelling need for neuroprotection of RGCs and optic nerve axons and as an additional therapeutic modality. The molecular changes occurring specifically in the RGCs, during the progression of glaucoma, contributing to neurodegeneration, are still poorly understood; hence identifying new therapeutic targets could provide more efficacious neuroprotective treatments.

ET-1 is a 21 amino acid vasoactive peptide that acts through two G-protein coupled receptors namely, ET_A_ and ET_B_ receptors, to produce diverse effects in various ocular tissues^8–11^. A growing body of evidence suggests that endothelins and their receptors are major contributors to neuronal damage in glaucoma^11–16^. The role of endothelins in glaucomatous neurodegeneration has been the subject of several review articles^12,17,18^. However, the detailed cellular and molecular mechanisms that contribute to ET-1 mediated-neurodegeneration are not completely understood. ET-1 concentrations were also found to be increased in several animal models of glaucoma^19–21^. Both intravitreal and peribulbar administration of ET-1 has been shown to produce axon loss, RGC loss and disruption of nerve fiber layer^11,22,23^.

Several studies point to the role of mitochondrial dysfunction and oxidative stress as contributors to glaucomatous damage in animal models of glaucoma^24–27^. There is an abundance of mitochondria in retinal ganglion cells (RGCs) within the soma region and the initial unmyelinated axons (anterior to the lamina cribrosa) of the optic nerve and synaptic terminals as opposed to the post-laminar and myelinated region. Hence, RGCs are typically more sensitive to mitochondrial dysfunction than other neuronal populations^28^. Retrograde and anterograde transport of mitochondria which occurs continuously along the axons of neurons, is vital for synaptic function. Hence, any disturbance of axonal transport due to mitochondrial abnormalities and a compromise in the mitochondrial function can lead to severe metabolic crises because of energy depletion^29^. The impairment in axonal transport can be observed commonly in diseases such as Amyotrophic Lateral Sclerosis and Leber’s Hereditary Optic Neuropathy^30^. Mitochondrial function decreases with age and as RGCs are largely dependent on their mitochondria, the prevalence of primary open angle glaucoma (POAG) increases with age possibly due to a decline in mitochondrial function^31^. Impaired mitochondrial dysfunction is a phenomenon observed in most, if not all, neurodegenerative disorders^32–35^. ET-1 elevates reactive oxygen species by acting through the ET_B_ receptor, which could be one mechanism by which ET-1 alters mitochondrial function^36^.

The effect of ET-1 on mitochondrial gene expression and function and their potential impact on neurodegeneration of RGCs has not been adequately explored. The purpose of this study was to use RNA-seq to investigate ET-1 mediated changes in gene expression of mitochondrial genes and proteins in primary RGCs treated with ET-1, as well as *in vivo* following either ET-1 administration or IOP elevation in rat eyes.

## Results

### Characterization, purity and yield of isolated primary RGCs

**Figure 1:**
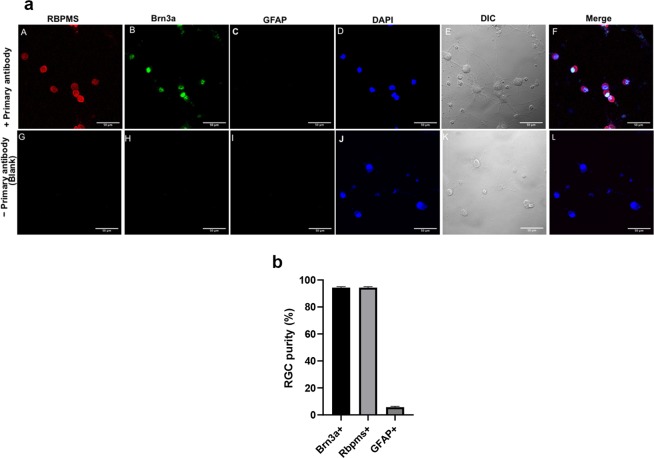
(**a**) Purity of primary RGC culture. Confocal representative images of pure RGC cultures immunolabeled with specific RGC markers, A) RBPMS (red), B) Brn3a (green) as well as with a specific marker for astrocytes/Muller cells, C) GFAP, while all isolated cells were stained with the D) nuclear dye DAPI (blue). A negative control immunostaining (Blank) in which the primary antibody was excluded showed no staining. Scale bar indicates 50 μm. (**b**) Quantitation of the RGC purity. Illustrated data are Mean±SEM from 3 independent experiments.

By sequential double immunopanning with the Thy1.1 antibody, we isolated approximately 20,000 to 40,000 RGCs per retina, with a yield of 40% to 50% of the RGCs in post-natal day 4–7 (P4-P7) rat retinas^35^. To confirm the purity of the RGCs, immunostaining using RGC specific markers, including, RBPMS (Fig. 1a) and Brn3a (Fig. 1b) was carried out and GFAP, an astrocyte marker was used as a negative control (Fig. 1c**)**. It was found that approximately 90 to 95% of the cells were immunopositive for RBPMS and Brn3a and hence were characterized as RGCs, whereas 2% of the cells were immunoreactive for GFAP. Thus, the immunopanning technique yielded a highly enriched RGC culture.

### Translatome analysis of primary RGCs following ET-1 treatment (100 nM)

**Figure 2:**
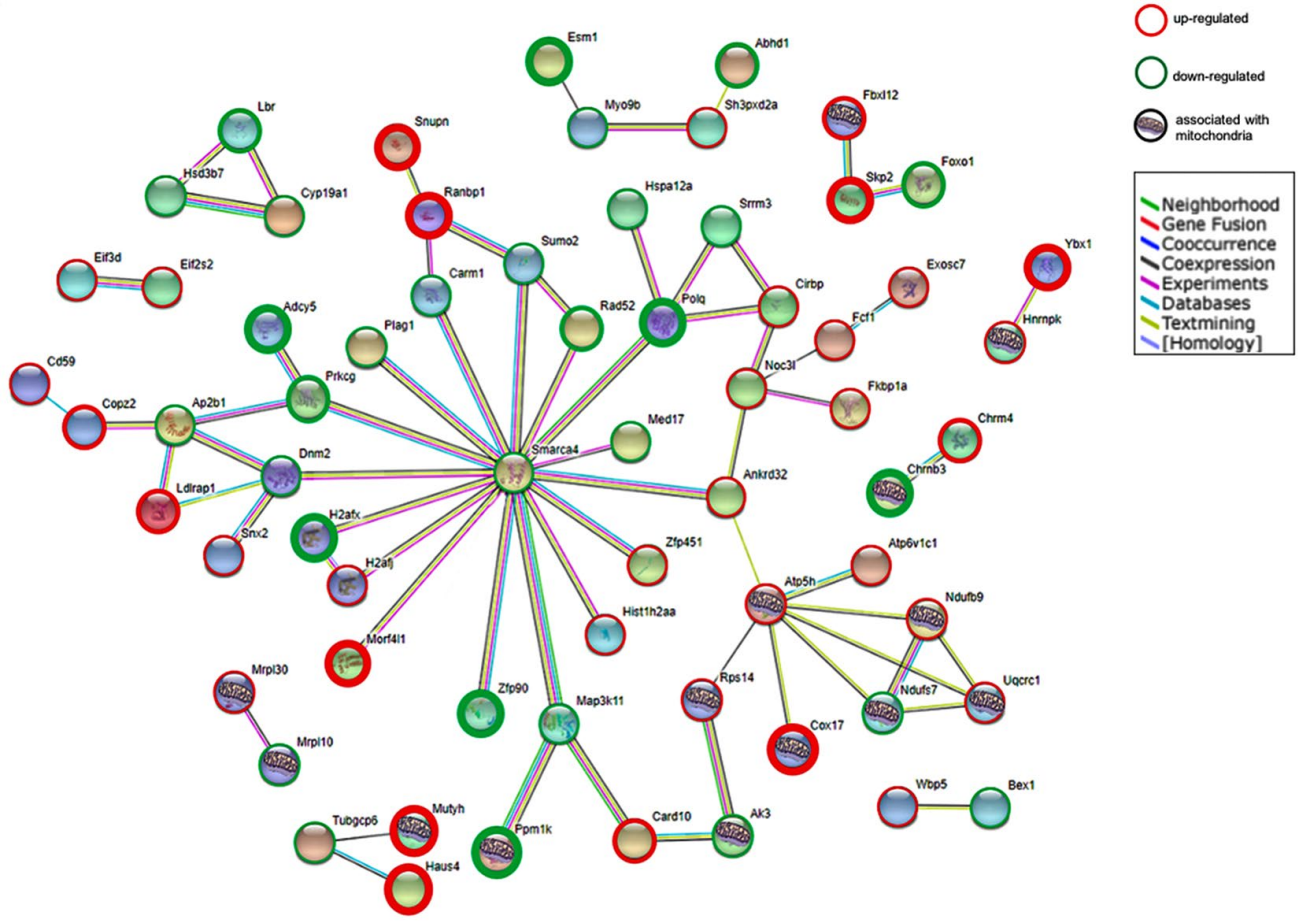
The DEGs in primary RGCs following ET-1 treatment for 24 hours were analyzed using the STRING database. The network nodes represent the proteins encoded by the DEGs. Seven different colored lines link a number of nodes and represent seven types of evidence used in predicting associations: green lines represent neighborhood evidence; red lines indicate the presence of fusion evidence; blue lines represent co-occurrence evidence; purple lines represent protein homology evidence; black lines represent co-expression evidence; pink lines represent experimental evidence; light blue lines represent database evidence; and yellow lines represent text-mining evidence.

Primary cultures of RGCs were isolated and treated with ET-1 for 24 h, following which polysomal RNA was isolated. RNA-seq analysis of polysomal RNA from untreated (control) and ET-1-treated RGCs was performed and bioinformatics analysis was carried out. From the STRING network analysis (Fig. 2), several mitochondrially-relevant genes which had a significant change in expression were identified. Gene Ontology (GO) classification with statistical overrepresentation tests indicate a significant enrichment in the differentially expressed genes (DEG) set for cellular components, including mitochondrion, endocytic vesicle membrane and cytoplasmic stress granules. The DEG set was also enriched for specific biological processes, including cation transport and metabolic processes. Twenty-three genes out of the 156 DEGs were identified to have a known or predicted mitochondrial function. Of the 23 genes, 14 of which are represented in the STRING network (indicated by the mitochondrial symbol on the node) are defined by some connected nodes. Four of the connected, mitochondrially-related genes were in the top 20 up-regulated and down-regulated genes in the list of 156 DEGs.

From the STRING network, mitochondrially-relevant genes were identified within the network and the direction of fold regulation was overlaid using red (up-regulated) and green (down-regulated) borders. It was found that 156 genes were differentially expressed following ET-1 treatment in RGCs. The thickness of the color indicator denotes the ranking of the gene: thick indicators are ranked in the top or bottom 20 of the ranked DEG list; medium borders are in the top / bottom 20 genes based only on magnitude of fold change; thin lines just indicate direction of fold change. Analysis of differential gene expression revealed a significant change in expression of several genes involved in mitochondrial function, oxidative metabolism and cell survival pathways. An increase in an expression of mitochondrial proteins cytochrome c oxidase copper chaperone (COX17) (3- fold), and ATP synthase, H^+^ transporting mitochondrial F0 complex (ATP5H) (3-fold) was found. On the other hand, a decrease in expression of glucose-6-phosphate dehydrogenase (5-fold), Forkhead box O1 (FOXO1) (986-fold), mitogen- activated protein kinase kinase kinase 11(Map3k11) (40-fold) and modulator of apoptosis (Moap1) (6255-fold) was observed.

### Translatome changes in primary RGCs following ET-1 treatment

**Figure 3:**
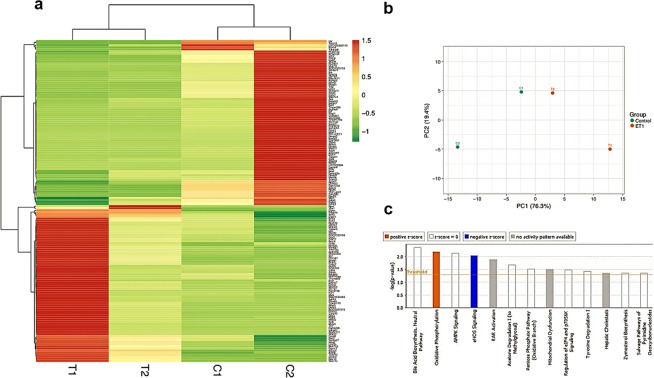
Correlation heatmap, Principal Component Analysis and Ingenuity Pathway Canonical Network Analysis. (**a**) Heatmap analysis, clustering of samples based on Pearson’s correlation showing hierarchical clustering of DEGs after ET-1 treatment. Rows representing genes are scaled, i.e., the value (z score) for a given gene in a given sample represents its deviation from the mean expression value of the gene across all samples in terms of its standard variation, with red denoting upregulation, and green downregulation. Each column represents a sample. Color key indicates relative levels of gene expression changes, with darker green indicating downregulation and darker red indicating upregulation. (**b**) PCA plot of samples shows the distribution of samples in the space of the first two components (PC1 and PC2). (**c**) Ingenuity Pathway Analysis of Canonical Pathway Enrichment. Z-score shows overall activation or suppression of the corresponding pathways.

To explore the translatome differences in primary retinal RGCs between the control and ET-1 treated samples, a heatmap was generated showing log2 (fold-change)> 1.5. Hierarchical clustering of the Pearson correlation coefficients was performed using the 156 DEGs to determine if the gene expression profiles were different between the two groups (Fig. 3a). The results showed that the replicates cluster together based on similarities in gene expression, although there was some variability observed in the top genes between the replicates. Further, two clear sets of genes with increased expression as well as genes with decreased expression following ET-1 treatment emerged. In addition, principal component analysis (PCA) of the normalized gene expression values for the top 156 DEGs showed that axes 1 and 2 accounted for 76.3% and 19.4% of the total variation, respectively (Fig. 3b). The sign (+/-) of PC1 distinguishes controls from ET-1 treated replicates, where PC1 is positive for ET-1 treatment and negative for the controls. Ingenuity Pathway Analysis (IPA) canonical pathway analysis of the gene set revealed that oxidative phosphorylation pathway is the top-most enriched pathway within the DEGs following ET-1 treatment (Fig. 3c).

### Validation of DEGs with qPCR

**Figure 4:**
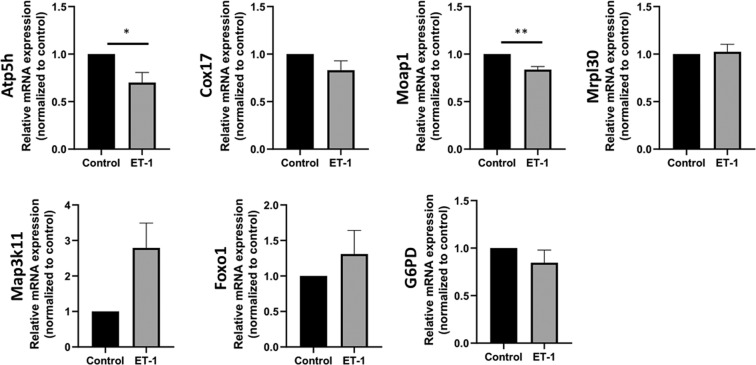
qPCR validation of the expression of selected genes related to mitochondrial function, neurodegeneration or cell survival. Primary RGCs were either untreated (control) or treated with ET-1 for 24 hours following which total cellular RNA was isolated and subjected to qPCR analysis of gene expression. Relative mRNA expression values are plotted as a ratio to untreated control levels. Data are expressed as Mean±SEM (n=3–4 biological replicates). (*p <0.05, **p ≤ 0.01).

To validate RNA-seq findings, qPCR analysis was performed on several selected genes associated with either mitochondrial function, neurodegeneration or cell survival. A significant decrease in gene expression of Atp5h (30%) and Moap-1 (17%) was observed following treatment of primary RGCs with ET-1 (Fig. 4). On the other hand, an increasing trend in the expression of FOXO1 (31%) and Map3K11 (179%) genes and a decreasing trend in the expression of Cox17 (17%) and G6PD (15%) genes were observed in ET-1 treated RGCs (Fig. 4). However, these were not statistically significant. The expression pattern observed by conventional qPCR was different in comparison to the RNA-seq data for some DEGs including Atp5h, Cox17 FoxO1, and Map3k11. There was no appreciable change in the expression of mitochondrial ribosomal protein L30 (Mrpl30) gene following ET-1 treatment when analysed by qPCR **(**Fig. 4**)**.

### ET-1 treatment causes a decline in key mitochondrial proteins COX17 and ATP5H in primary RGCs

**Figure 5:**
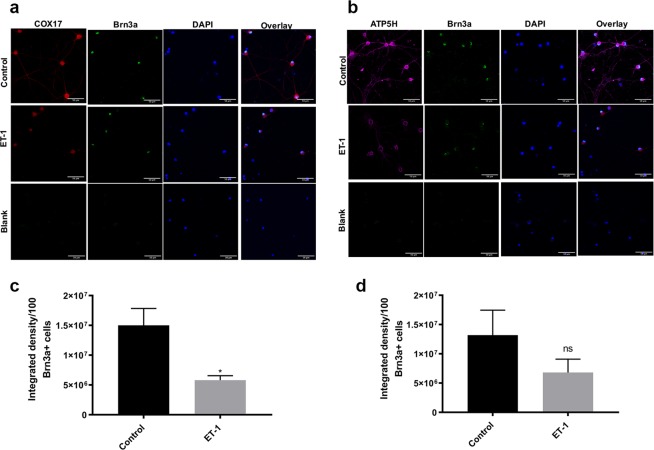
Immunofluorescence analysis of ATP5H and COX17 in control and ET-1 treated RGCs. Immunostaining for (**a**) COX17 (red) and (**b**) ATP5H (magenta) in primary RGCs. Scale bar, 50 μm and quantitation of COX17 and ATP5H (**c,d**) from five different images (n = 5) following ET-1 treatment. Quantitation of fluorescence intensity of COX17 and ATP5H calculated as integrated density/Brn3a positive 100 cells as determined from the five different images. Data are expressed as Mean±SEM (*P < 0.05).

Mitochondrial dysfunction and oxidative damage are associated with many neurodegenerative disorders including glaucoma. Hence, we focused our attention on the expression of some mitochondrial genes that are important components of the mitochondrial oxidative phosphorylation pathway. To observe changes at the protein level, immunostaining for ATP5H and COX17 was carried out (Fig. 5a,b). Staining for both ATP5H and COX17 was observed both in the soma and axonal processes of RGCs.

Immunocytochemical analysis revealed that ET-1 treatment produced a decrease in immunostaining of key mitochondrial proteins ATP5H and COX17, indicating that ET-1 has the potential to compromise the bioenergetics of RGCs. Quantitation of the immunofluorescence of both ATP5H and COX17 revealed a decrease in integrated density/100 Brn3a positive cells following ET-1 treatment. (Fig. 5c,d) However, immunostaining of only COX17 showed a significant (p<0.05) decrease in integrated density/100 Brn3a positive cells.

### A significant decrease in mitochondrial rate of ATP production was observed following ET-1 treatment in primary RGCs

**Figure 6:**
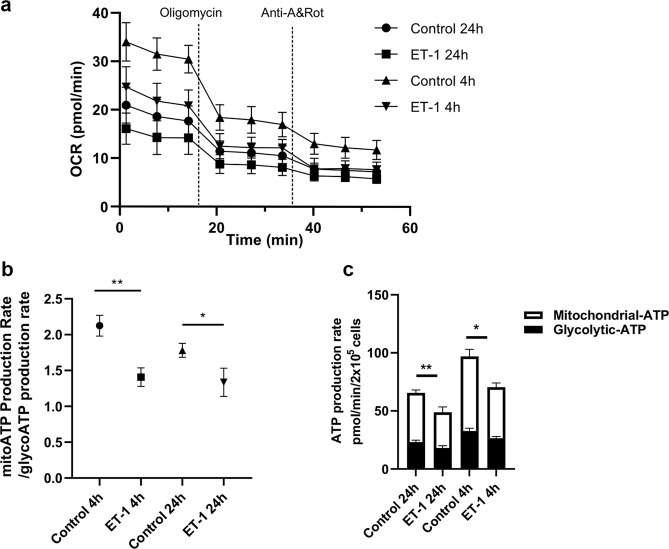
Quantification of ATP production by Seahorse XF real-time ATP rate assay following ET-1 treatment at 4 h and 24 h in primary RGCS. (**a**) Kinetic profile of OCR measurements following ET-1 treatment at 4 h and 24 h. (**b**) ATP rate index indicating the changes in metabolic phenotype after ET-1 treatment at 4 h and 24 h. An increase in the XF ATP Rate Index represents a more oxidative / less glycolytic phenotype and vice-versa. (**c**) Metabolic flux analysis showing quantification of mitochondrial ATP production and glycolytic ATP production. Data shown are Mean±SEM (n=4 biological replicates and atleast 6 technical replicates), *p <0.05, **p ≤ 0.01).

Since a decrease in immunostaining for ATP5H and COX17 was found in RGCs treated with ET-1, a Seahorse XF ATP rate assay was carried out to determine if a functional decline in mitochondrial ATP production was observed. As seen in Fig. 6a, the OCR differed at the 4 h and 24 h timepoints of ET-1 treatment. At the 4 hour time point, RGCs had a higher basal level of OCR (35 pmol/min), compared to the 24 hour time point (20 pmol/min). At both 4 h and 24 h time points of ET-1 treatment, the OCR was reduced, compared to the corresponding untreated controls. ET-1 treatment produced a significant decrease in the rate of mitochondrial ATP production at both 4 h and 24 h time points following treatment with 100 nM ET-1 (Fig. 6b). The glycolytic ATP production rate showed a significant decrease following 4 h and a decreasing trend at 24 h following ET-1 treatment in RGCs. When the ratio of the mitochondrial to glycolytic ATP production was calculated, a significant decrease of the ratio was found at both 4 h and 24 h time points of ET-1 treatment (Fig. 6c).

### Decrease in expression of mitochondrial proteins COX17 and ATP5H following intravitreal administration of ET-1

To determine if ET-1-mediated changes in ATP5H and COX17 were also observed *in vivo*, intravitreal injection of ET-1 was carried out in one eye of Brown Norway rats. The contralateral eye was injected with the vehicle used to dissolve ET-1. Rat retina section from vehicle-injected rat eyes showed immunoreactivity for ATP5H and COX17, which was assessed in multiple retinal layers including the nerve fiber layer (NFL), ganglion cell layer (GCL) and inner plexiform layer (IPL).

**Figure 7:**
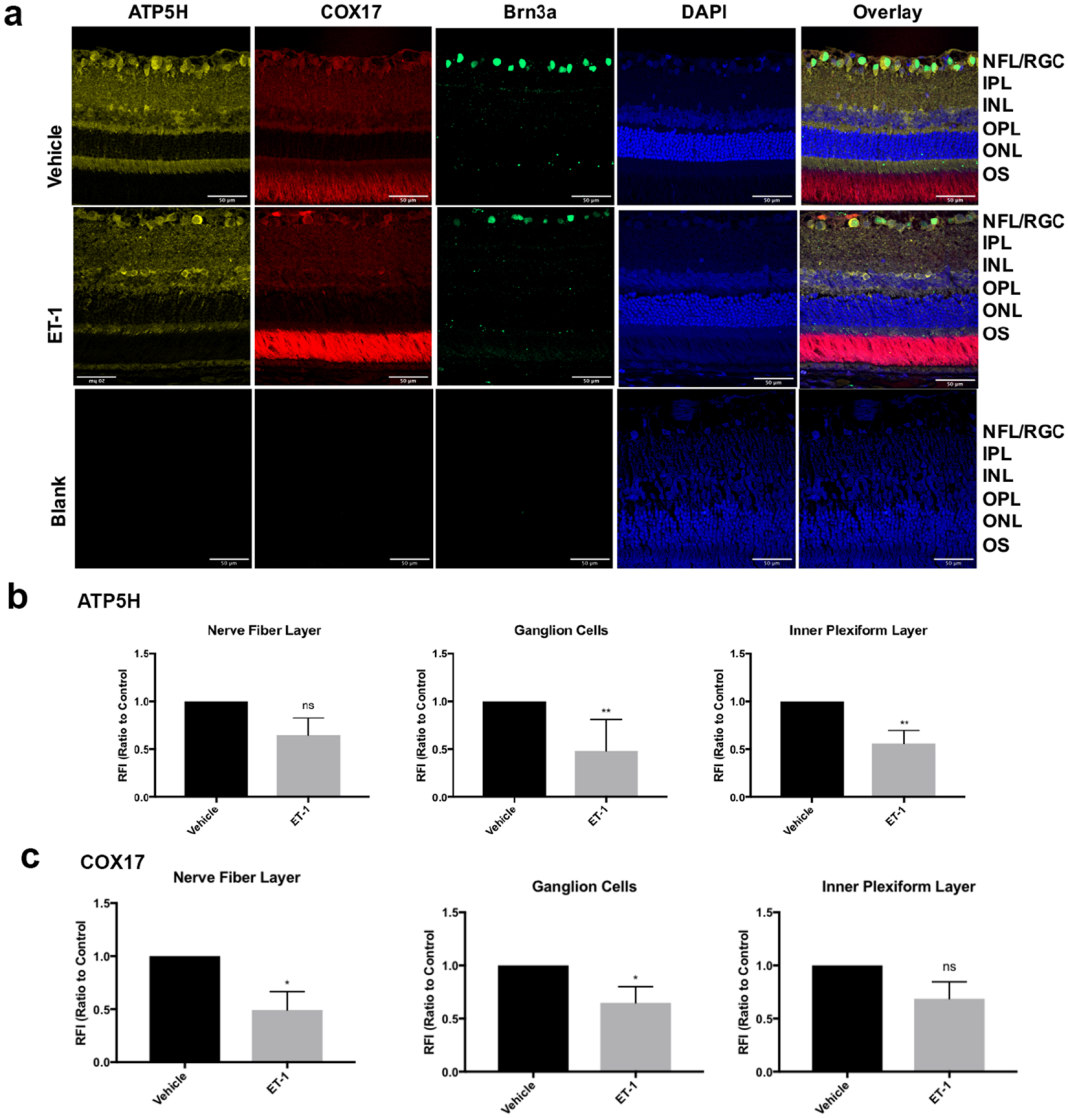
ATP5H and COX17 expression in retinas of adult Brown Norway rats following intravitreal administration of ET-1. (**a**) Representative images. Immunostaining of retina sections probed for ATP5H (yellow) and COX17 (red) following ET-1, 500 (μM) intravitreal injection 24 hours. Scale bar, 50 μm. Relative fluorescent intensity for ATP5H (**b**) and COX17 (**c**) for the NFL; GC and IPL. Bars represent mean ± SEM (n = 6 animals/group). Asterisks indicate statistical significance *p <0.05; **p <0.01; by student’s t-test. ONL outer nuclear layer, OPL outer plexiform layer, INL inner nuclear layer, IPL inner plexiform layer, GCL ganglion cell layer, NFL nerve fiber layer.

In the vehicle treated eyes, robust immunostaining for ATP5H was detected in the retinal ganglion cells, inner plexiform layer and outer plexiform layer (Fig. 7a). On the other hand, immunostaining for COX17 was found predominantly in the nerve fiber layer, retinal ganglion cells and inner plexiform layer in vehicle treated rat eyes (Fig. 7a). A semi-quantitative analysis measuring the change in integrated intensity was also performed using ImageJ software. Projections from confocal image z-stacks were analyzed for integrated density of ATP5H and COX17 expression in ET-1 treated and vehicle-treated eyes to determine fold change in expression. Compared to the vehicle-treated controls, retinas obtained from ET-1 treated eyes showed a statistically significant decrease in ATP5H expression mainly in the GCL (RFI = 0.4818 ± 0.1328, n = 6, p<0.01) and IPL (RFI = 0.5633 ± 0.1333, n = 6, p < 0.01) (Fig. 7b). Expression of ATP5H was co-localized with Brn3a (a RGC specific marker) in RGCs (Fig. 7b). Additionally, a modest decrease in immunostaining for ATP5H was also observed in the NFL compared to the vehicle-control. (RFI = 0.645 ± 0.1814, n = 6).

Similar to the pattern of expression observed for ATP5H, ET-1 treatment (24 h) showed decreased immunoreactivity for COX17 in NFL, GCL and IPL in rat retinas (Fig. 7c). A semi-quantitative analysis performed indicated a statistically significant decrease in COX17 expression in the NFL (RFI = 0.4914 ± 0.1738, n = 6, p <0.05) and GCs (RFI = 0.6483 ± 0.1518, n = 6, p <0.05) (Fig. 7c). A statistically significant difference was not found in the IPL (RFI = 0.6867 ± 0.1593, n = 6), however, the same trend of decrease as observed in ATP5H expression was still present. Taken together, the data suggest that there was a downregulation in the expression of the key mitochondrial proteins ATP5H and COX17, 24 hours following ET-1 treatment.

### IOP elevation produced a modest decrease in immunoreactive levels of ATP5H

**Figure 8:**
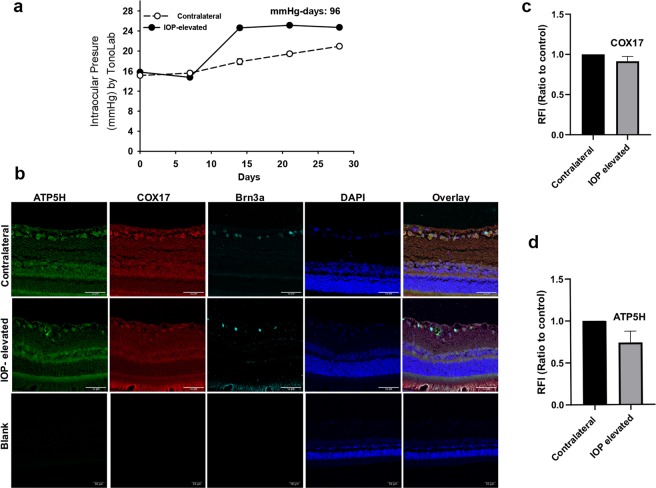
ATP5H and COX17 expression in Morrison’s model of ocular hypertension following 2 week IOP elevation. (**a**) Representative graph of IOP measurements for IOP elevated (white circles) and contralateral control (black circles) eyes in adult female Brown Norway rats. (**b**) Representative confocal microscopy images. Immunostaining of retina sections probed for ATP5H (green), COX17 (red), Brn3a (cyan) following 2 weeks of IOP elevation. C) Quantitation of the Relative fluorescent intensity for (**c**) COX17 and D) ATP5H estimated by quantifying only Brn3a positive cells. Scale bar = 50 μm. Bars represent Mean ± SEM (n = 3 animals/group).

Retina sections were obtained from four retired breeder Brown Norway rats, which were IOP elevated in one eye, using the other eye as the contralateral control (Fig. 8a). Immunostaining was carried out using antibodies to ATP5H and COX17 and images were taken on a Zeiss LSM580 confocal microscope. A semi-quantitative analysis measuring the change in integrated intensity was also performed using ImageJ software. Projections from the confocal image z-stacks were analyzed for integrated density of ATP5H and COX17 immunostaining and compared between the IOP-elevated eyes and their corresponding contralateral eyes (Fig. 8b). It was found that IOP elevation produced a decreasing trend in immunostaining for ATP5H, compared to that of the corresponding contralateral eyes (Fig. 8d). There was no appreciable change in immunostaining for COX17 between IOP elevated eyes and contralateral eyes (Fig. 8c).

## Discussion

ET-1 is a potent vasoactive peptide that has been shown to be elevated in both the aqueous humor and circulation of primary open angle glaucoma patients^37–40^. An elevation of both ET-1 and homocysteine was found in the plasma of primary open angle glaucoma patients, compared to normal tension glaucoma as well as control subjects, suggesting that elevated oxidative stress and ET-1 are involved in the early stages of glaucoma pathology^41^. Single nucleotide polymorphisms (SNPs) in both the ET-1 and ET_A_ receptor genes that affected ET-1 plasma concentrations and higher ET-1 levels correlated with increased visual field damage^42^. A recent meta-analysis that included seven studies (212 cases, 164 controls) for normal tension glaucoma and six studies (160 cases, 174 controls) for the POAG analysis demonstrated increased circulating ET-1 levels in glaucoma patients, compared to control subjects^43^.

Several studies have pointed to ET-1’s key role in neurodegeneration, however, the precise mechanisms by which ET-1 produces neurodegenerative effects is still an active area of investigation. Previous studies from our laboratory have shown that intravitreal ET-1 injection in rats decreased anterograde axonal transport of vesicles associated with the mitochondrial subcomponent^9^. Axonal transport is an energy dependent process, mediated by motor proteins which use the energy of ATP hydrolysis to produce force and movement along the microtubules. The decrease in fast axonal transport could be due to a decline ATP production, disruption of the cytoskeleton or a combination of these factors. Most of the ATP needed for cellular metabolism/activities is generated by oxidative phosphorylation in the mitochondria. This raises the possibility that ET-1 could alter mitochondrial dynamics as well as oxidative metabolism in the optic nerve head during glaucoma. It has been shown that ET_B_ receptor stimulation by administration of sarafotoxin (ET_B_ receptor agonist) in rats increases the production of superoxide anions, in sympathetic neurons^36^. Increased oxidative stress by endothelin receptor stimulation could have damaging effects on the mitochondria, thereby providing a strong rationale to assess expression of mitochondrial genes and their contribution to neurodegeneration.

Dysregulation of gene expression contributes to neurodegeneration in glaucoma, by acting on several cellular pathways, ultimately enhancing pro-apoptotic effects while suppressing anti-apoptotic gene expression^44,45^. Numerous studies have been carried out using total cellular RNA from either the retina or cultured RGCs to evaluate changes in gene expression in various models of optic neuropathy^46,47^. While an assessment of changes in gene expression at the mRNA level is informative, it does not provide an indication of the corresponding changes in the protein levels. In the polysomal profiles, there is a drastic shift of distribution of mRNA and its association with multiple ribosomes, following metabolic insults before it is detected on the proteome level^48^. Hence, assessment of polysomal RNA will provide a reflection of the translatome, offering a glimpse into *de novo* protein synthesis which is an important manifestation of changes in gene expression at the protein level.

In the current study, using RNA-seq we analyzed the changes in gene expression of polysomal RNA which represent the pool of actively translated mRNAs. This provides a unique opportunity to identify novel genes whose expression are altered at the protein level during pathogenesis. Identification of novel genes that contribute to glaucomatous neurodegeneration will provide the basic information needed to develop new therapies aimed at neuroprotection in glaucoma. To our knowledge, this is the first study to use RNA-seq analysis to investigate the RGC translatome profile following ET-1 treatment. Although other researchers have conducted microarray analyses of gene expression under glaucomatous conditions, most of these studies focused on mRNA expression which may not be reflected at the level of the protein expression^49–51^.

The results of the RNA seq analysis were confirmed by qPCR analysis of some of the candidate genes. Some of the data derived from the qPCR data were different from that found in the RNA-seq analysis. For instance, while an increase in Atp5h and Cox17 was found in the RNA-seq analysis, a decrease in the expression of these genes were found by qPCR analysis. Some of these variations could be related to the template half-life of the individual transcripts. Less abundant transcripts in RNA samples with low concentrations could be rapidly degraded, particularly with longer handling times. The differences in the handling time between RNA-seq analysis and qPCR analysis could be one plausible reason for the discrepancies in the findings between RNA-seq and qPCR, as well as account for the big variations observed in RNA expression that contributed to the lack of statistical significance in many of the qPCR analyses. On the other hand, at the protein level, the data was very consistent and a decrease in levels of ATP5H was observed both in cultured RGCs treated with ET-1 and in retinas of rats intravitreally injected with ET-1.

A notable finding from the RNA-seq analysis was that 23 out of 156 DEG in the RNA-seq analysis were identified with known or predicted mitochondrial function. Ingenuity Pathway Analysis revealed oxidative phosphorylation as the top significantly enriched canonical pathway, which points to the involvement of mitochondrial bioenergetics in RGCs with ET-1 treatment. To specifically assess the nuclear encoded genes in the mitochondrial respiratory chain complex and oxidative phosphorylation, the mRNA and protein expression of Cox17, and Atp5h which are critical components of Complex IV and Complex V, respectively, were determined. COX17 is the terminal enzyme of the mitochondrial respiratory chain while mitochondrial ATP synthase catalyzes ATP synthesis, utilizing an electrochemical gradient of protons across the inner membrane during oxidative phosphorylation^28,52^. IOP elevation has previously shown to cause a reduction of Cytochrome c oxidase IV subunit 1 activity by damaging the mitochondria in response to mitochondrial fission^53^. There is a decline of mitochondrial function during early stages of POAG due to reduction in ATP synthesis, particularly making RGCs susceptible for oxidative phosphorylation impairment^54^. Mitochondrial mutation and mitochondrial genetic variation can also act as a potential risk factor in POAG pathogenesis^55,56^. Interestingly, a recent study showing association of mitochondrial proteins with the pathogenesis of POAG identified a number of key genes including Atp5h, Cox17, Ndufs7, Ndufb9, Uqcrc1 and Ak3 involved in oxidative phosphorylation in human POAG that were identical to some of the genes observed in our findings^57^. In the current study, we identified that there was a decline in two important mitochondrial proteins ATP5H and COX17 both in primary RGCs treated with ET-1 as well as in rats intravitreally injected with ET-1. In addition, a decreasing trend in immunostaining for ATP5H was found following IOP elevation in Brown Norway rats.

The IOP elevation model is a chronic model and the data presented is indicative of IOP-mediated changes that occur over longer period of time, compared to the acute effects that occur following a bolus intravitreal administration of 2 nmole of ET-1. Nevertheless, a trend towards a decrease in ATP5H was observed following IOP elevation for 2 weeks in Brown Norway rats. This suggests that a decline in mitochondrial function could compromise bioenergetics mediated by a decline in ATP levels. The Seahorse assay which exploits the coupling of ATP synthesis to oxygen consumption that occurs during oxidative phosphorylation, provided a clear evidence for the ability of ET-1 to significantly decline mitochondrial ATP production in RGCs. The use of oligomycin to inhibit mitochondrial ATP synthase and assess the decline in the oxygen consumption rate, provides a glimpse of ATP-linked respiration, which differed significantly between the control and the ET-1 treated RGCs. The data points to the ability of ET-1 to functionally impact ATP synthesis in RGCs. The findings have important implications for RGC death occurring through energy depletion, which could occur through opening of permeability transition pore, leading to mitochondrial depolarization and mitochondrial damage. It is well known that reactive oxygen species are a major contributor to mitochondrial outer membrane permeabilization. As mentioned earlier, ET_B_ receptor activation has been shown to result in elevation of oxidative stress as reported in dorsal root ganglion neurons^36^. It is plausible that similar mechanisms could operate to generate oxidative stress through the actions of ET-1 on its receptors in RGCs.

Taken together, our findings have important implications for the role of mitochondria in the pathogenesis of glaucoma. Further work is required to understand the role of the genes identified in the RNA-seq analysis and elucidate the signaling pathways which contribute to the expression of these genes. The current study could provide an insight to advance our understanding of mitochondrial dynamics contributing to RGC loss in POAG.

## Methods

### Isolation of primary retinal ganglion cells

Primary rat RGCs isolation was performed according to the method of Barres *et al*.^58^. All protocols and procedures were in accordance with the policies of the Association for Research in Vision and Ophthalmology (ARVO) for use of animals in research and approved by the Institutional Animal Care and Use Committee (IACUC) (animal protocol #IACUC-2017-0024) at University of North Texas Health Science Center at Fort Worth, TX, USA. Post-natal day 4–7 rat pups were euthanized, and the isolated retinas were treated with 4.5 units/ml of papain solution to dissociate the cells. The cell suspension was subsequently incubated for 10 minutes with a rabbit anti-macrophage antibody and transferred to a petri dish coated with a goat anti-rabbit IgG (H + L chain) antibody for 35 minutes. Cells that were not attached to the coated anti-rabbit IgG were transferred to a petri-dish coated with anti-Thy1.1 antibody for 1 hour with intermittent shaking. Cells were then detached by trypsin treatment and collected by centrifugation. Cells were seeded either directly in a 6-well plate or on coverslips coated with poly-D lysine and mouse-laminin and cultured in serum-free Dulbecco’s modified Eagle’s medium containing brain-derived neurotrophic factor (BDNF) (50 ng/ml; Peprotech, Rocky Hill, NJ, USA), ciliary neurotrophic factor (10 ng/ml; Peprotech), and forskolin (5 ng/ml; Sigma-Aldrich Corp.). Cells were incubated at 37 °C in a humidified atmosphere of 10% CO_2_ and 90% air. One-third volume of the culture medium was changed every two days. The purity of the culture was determined by counting RBPMS and Brn3a positive cells per 100 cells from different regions in an image. The purity of the culture obtained is routinely between 90%-95% in most batches of isolated RGCs.

### ET-1 treatment

ET-1 peptide was purchased from Bachem (Torrance, CA) and dissolved to a stock concentration of 500 μM and working stocks of 100 μM. The seeding density of the primary RGCs was approximately 350,000 / well in a 6-well plate. After 1 week in culture to promote neurite outgrowth, the isolated RGCs were treated with 100 nM ET-1 (final concentration) for 24 hours while untreated RGCs served as a control.

### Polysomal RNA isolation

Following ET-1 treatment, a brief incubation with cycloheximide (10 μg/ml) to inhibit protein synthesis was done, total polysomes were isolated by magnesium precipitation by a modification of the method of Palmiter *et al*.^59^. Cells were washed, scraped in PBS and the cell pellet was suspended in 150 μl of homogenization buffer (25 mM Tris-HCl pH 7.5, 25 mM NaCl, 5 mM MgCl_2_, and 1% Triton X-100). After an incubation on ice for 5 min, the homogenate was centrifuged at 10,000xg in a microfuge for 10 min at 4 °C to sediment the nuclei and mitochondria. The pellet was discarded and the supernatant was treated with an equal volume of polysome precipitation buffer (25 mM Tris-HCl pH 7.5, 25 mM NaCl, 250 mM MgCl_2_ and 1 M sucrose). The content of the tubes were mixed by inverting and incubated on ice for one hour with intermittent mixing. The polysomal fraction was obtained by centrifugation at 40,000 × g for 1 hr in a Beckman ultracentrifuge at 4 °C using the TLA-55 fixed angle rotor. The supernatant was discarded and the polysomal pellet was extracted using the Trizol reagent to isolate polysomal RNA according to the manufacturer’s instructions.

### Library preparation and RNA-seq analysis

Libraries for RNA-Seq were prepared in the UCLA core facility with Clonetech SMARTer Stranded Total RNA-Seq (Pico) Kit. The workflow consisted of first-strand synthesis, template switching, adaptor ligation, cleavage of ribosomal cDNA and PCR amplification. Different adaptors were used for multiplexing samples in one lane. Sequencing was performed on Illumina Hiseq. 3000 for a single read 50 bp run. Data quality check was done on Illumina SAV. The reads were first mapped to the latest UCSC transcript set using Bowtie2 version 2.1.0^60^ and the gene expression level was estimated using RSEM v1.2.15^61^. TMM (trimmed mean of M-values) was used to normalize the gene expression. Differentially expressed genes were identified using the edgeR program^62^. Genes showing altered expression with p<0.05 and more than 1.5 fold changes were considered differentially expressed, since a fold increase of less than 1.5-fold would typically not produce appreciable phenotypic changes. Differentially expressed genes (DEGs) based on grouped comparison of untreated and treated cells were analyzed for biological and functional relevance using the *Rattus norvegicus* knowledgebase in PANTHER v.13.1; GO classifications based on cellular component, molecular function, biological process, and reactome pathways were analyzed. MitoMiner v.4.0 was used to identify the DEG subset that has a known or predicted mitochondrial function. A STRING network was constructed using the 156 genes to identify known and potential protein-protein interactions within the set of differentially expressed genes (DEGs). DEGs were ranked using the sign (fold change)*(1/p-value) method to identify genes that are both highly dysregulated (up/down fold change magnitude) and with a low probability of false discovery (low p-value).

### Quantitative real-time PCR

Primary RGCs were isolated from post-natal day 4–6 rat pups (as described above) and total cellular RNA was extracted using the Trizol reagent (as described above). Complementary DNA was prepared from total cellular RNA using iScript Reverse Transcription Kit (BioRad, Hercules, CA), and Real-time PCR (SsoAdvanced™ SYBR^®^ Green Supermix) was performed using cDNA as template to detect gene expression of some DEGs including Mitochondrial Ribosomal Protein L30 (Mrpl30), ATP Synthase, H^+^ Transporting, Mitochondrial F0 Complex, Subunit D (Atp5h), cytochrome C oxidase copper chaperone (Cox17), Glucose-6-phosphate dehydrogenase (G6pd), Forkhead box protein (Foxo1), Mitogen-Activated Protein Kinase Kinase Kinase 11 (Map3k11) and Modulator Of Apoptosis 1 (Moap1). PCR conditions were as follows: 95 °C for 3 minutes followed by 40 three-step cycles of 95 °C for 10 seconds, 60 °C for 30 seconds and 72 °C for 30 seconds. PCR primers were validated and the authenticity of the amplicons were confirmed by their melting temperatures. RNA samples from 3–4 biological replicates were used for real-time PCR analysis. Data was expressed as mean ± SEM and normalized to cyclophilin-1. The results were presented as relative fold change compared to untreated control. The statistical significance was calculated by Student’s *t*-test, and *p* values <0.05 were considered significant.

### Immunocytochemistry

Purified RGCs were cultured for 7 days to promote neurite outgrowth. RGCs were then treated with 100 nM ET-1 for 24 h and fixed with 4% paraformaldehyde for 10 min. After permeabilization with 0.1% Triton X-100, the cells were blocked with 5% bovine serum albumin and 5% normal donkey serum at room temperature for 1 hour to prevent non-specific binding with the secondary antibody. Blocking was followed by overnight incubation at 4 °C with primary antibodies against mitochondrial proteins ATP5H and Cox17. Primary antibodies used were rabbit anti-COX 17 (1:100, #ab69611, Abcam), mouse anti-ATP5H (1:200, #ab110275, Abcam) and goat anti-Brn3a (1:200, #sc31984, Santa Cruz Biotechnology). Following primary antibody incubations, cells were washed with PBS and then incubated with the corresponding secondary antibody at room temperature (RT) in the dark. Secondary antibodies used were donkey anti-rabbit Alexa 647 (1:1000, Invitrogen), donkey anti-mouse Alexa 546 (1:1000, Invitrogen) and donkey anti-goat 488 (1:1000, Invitrogen). Immunostaining carried out with the exclusion of the primary antibody and using only the secondary antibody conjugated to the fluorophore served as the negative control, which was indicative of non-specific staining by the secondary antibody. Following the secondary antibody incubation, cells were washed with PBS and incubated with with 4′ 6 Diamidino-phenylindole dichloride (DAPI) to stain cell nuclei. The coverslips were mounted on slides with antifade medium (FluorSave; Calbiochem, La Jolla, CA, USA). Images were captured for each field of view using the Zeiss 510 meta confocal microscope.

### Intravitreal injection of ET-1

ET-1 peptide was purchased from Bachem (Torrance, CA) and dissolved to a stock concentration of 500 μM. One group of male Brown Norway rats (n=6) were intravitreally injected in one eye with 4 μl of 500 μM ET-1 in 0.25% glacial acetic acid (adjusted to pH 7.0) while the contralateral eye served as control. The other group (n=6) received vehicle (0.25% glacial acetic acid, neutralized to pH 7.0) intravitreally in one eye, while the contralateral eye was control. Rats were euthanized 24 hours after injection by intraperitoneal pentobarbital injection (120 mg/kg body wt) followed with intracardiac pentobartital injection.

### Morrison model of IOP elevation in rats

IOP elevation was carried out in one eye of four retired breeder female Brown Norway rats by injection of 100 μl of hypertonic saline through episcleral veins^63^. Briefly, rats were anesthetized by inhalation of isoflurane and ensured by lack of a toe pinch reflex. Following a conjunctival incision, hypertonic saline (1.8 M NaCl) was injected into an episcleral vein using a glass needle (TIP01TW1F, WPI). The companion eye was used as the contralateral control eye. A flow rate of 309 µL/min was maintained during the injection for 10 to 20 seconds, thereby injecting 50 to 100 μl of hypertonic saline into the episcleral veins. IOP was measured two times a week using a hand-held TonoLab tonometer (iCare, Finland). Six IOP readings were averaged from each IOP measurement and ten IOP measurements were obtained for each eye. IOP plots were generated from IOP values obtained from the surgically treated eye and contralateral control eye. The IOP exposure in each rat was computed by the integral product of the extent of IOP elevation and the number of days of IOP elevation (expressed as mm Hg-days).

### Immunohistochemistry

Eyes were enucleated and a small incision (4 mm) was made posterior to the limbus and the eye was fixed in 4% paraformaldehyde (PFA) in phosphate buffered saline (PBS) for 30 min. Subsequently, an incision was made along the entire circumference of the limbus to remove the entire anterior segment. The optic cups were fixed in 4% PFA for an additional 3 hours. After fixation, the tissue was rinsed with 70% ethanol and later embedded in paraffin. Seven-micron sagittal retinal sections through the optic nerve head were obtained, de-paraffinized in xylene, and rehydrated with a graded series of ethanol. Following permeabilization with 0.1% Triton X-100 and blocking with 5% normal donkey serum containing 5% BSA in PBS, retinal sections were incubated with the appropriate primary antibodies overnight. Primary antibodies used were rabbit anti-Cox 17 (1:100, #ab69611, Abcam), mouse anti-ATP5H (1:200, #ab110275, Abcam)and goat anti-Brn3a (1:200, #sc31984, Santa Cruz Biotechnology). Secondary antibody incubation for 1 hr was carried out at room temperature. Secondary antibodies used were either donkey anti-rabbit Alexa 546 (1:1000, Invitrogen), donkey anti-mouse Alexa 647 (1:1000, Invitrogen) or donkey anti-goat 488 (1:1000, Invitrogen). Retinal sections incubated with no primary antibody served as the blank to assess non-specific staining by the secondary antibodies.

### Seahorse XF real-time ATP rate assay

Agilent Seahorse XFe96 analyzer was used to measure multiple parameters including oxygen consumption rate (OCR), total ATP production by quantifying ATP production rate from both glycolytic and mitochondrial pathways and ATP rate index. Briefly, primary RGCs were isolated from post-natal day 4–6 rat pups. The RGCs were seeded RGC medium in a poly-D-lysine coated seahorse plate and maintained in culture for one week to allow neurite outgrowth. The RGCs were either untreated or treated with ET-1 for 4 h or 24 h in trophic factor-free medium. A day prior to the experiment seahorse sensor cartridge was hydrated with water and incubated in a non CO_2_ incubator at 37 °C. Two hours before the experiment, the water was replaced with a seahorse calibrant. On the day of the experiment, seahorse XF DMEM (pH=7.4) was supplemented with 1 mM pyruvate, 2 mM glutamine and 10 mM glucose (Agilent, USA). The RGC medium was then replaced with 180 µl of Seahorse XF base medium and the cells incubated in a non CO_2_ incubator at 37 °C for 1 hour. During this incubation period, oligomycin and Rotenone/antimycin (Agilent, USA) were prepared in the seahorse medium to achieve final concentrations of 1.5 μM and 0.5 μM respectively when injected. From these stock solutions, 20 μl and 22 μl of Oligomycin and rotenone/antimycin respectively were then loaded into the drug delivery ports A and B of the hydrated sensor cartridge and loaded into the seahorse XF analyzer to calibrate for 30 minutes. The calibration plate was replaced with the cell culture plate and oxygen consumption (OCR) and extracellular acidification rate (ECAR) was monitored following the sequential injection of Oligomycin and rotenone/antimycin with each cycle set as 3 min mix, 2 min delay and measure for 3 minutes. According to the manufacturer’s instructions (Agilent Technologies, Santa Clara, CA), the following equation was used to calculated mitochondrial ATP production:

$${{\rm{OCR}}}_{{\rm{ATP}}}({\rm{pmol}}\,{{\rm{O}}}_{2}/{\rm{\min }})={{\rm{OCR}}}_{{\rm{Untreated}}}({\rm{pmol}}\,{{\rm{O}}}_{2}/{\rm{\min }})-{{\rm{OCR}}}_{{\rm{Oligomycin}}}({\rm{pmol}}\,{{\rm{O}}}_{2}/{\rm{\min }}).$$

The ATP production rate in ET-1 treated RGCs was compared to that of untreated RGCs for two time points of ET-1 treatment (4 h and 24 h). Data was normalized to the cell number of each well by Calcien AM assay.

### Statistical analysis

GraphPad Prism 7 (La Jolla, CA, USA) was used to perform statistical analysis. Student’s t-test was used while comparing two experimental groups (control versus treated). Statistical significance of the experimental data was described as *p < 0.05; **p < 0.01. Data are presented as mean ±SEM.
